# Vitamin K Supplementation for Prevention of Vascular Calcification in Chronic Kidney Disease Patients: Are We There Yet?

**DOI:** 10.3390/nu14050925

**Published:** 2022-02-22

**Authors:** Stefanos Roumeliotis, Anila Duni, Vasilios Vaios, Athanasios Kitsos, Vassilios Liakopoulos, Evangelia Dounousi

**Affiliations:** 1 Division of Nephrology and Hypertension, 1st Department of Internal Medicine, AHEPA Hospital, School of Medicine, Aristotle University of Thessaloniki, 54636 Thessaloniki, Greece; st_roumeliotis@hotmail.com (S.R.); vvaios_85@yahoo.gr (V.V.); vliak@auth.gr (V.L.); 2 Department of Nephrology, Faculty of Medicine, School of Health Sciences, University of Ioannina, 45110 Ioannina, Greece; anikristduni@yahoo.com (A.D.); thkitsos@hotmail.com (A.K.)

**Keywords:** cardiovascular disease, chronic kidney disease, end-stage kidney disease, hemodialysis, Matrix Gla Protein, menaquinone-7, vascular calcification, vitamin K

## Abstract

Chronic Kidney Disease (CKD) patients are at high risk of presenting with arterial calcification or stiffness, which confers increased cardiovascular mortality and morbidity. In recent years, it has become evident that VC is an active process regulated by various molecules that may act as inhibitors of vessel mineralization. Matrix Gla Protein (MGP), one the most powerful naturally occurring inhibitors of arterial calcification, requires vitamin K as a co-factor in order to undergo post-translational γ-carboxylation and phosphrorylation and become biologically active. The inactive form of MGP (dephosphorylated, uncarboxylated dp-ucMGP) reflects vitamin K deficiency and has been repeatedly associated with surrogate markers of VC, stiffness, and cardiovascular outcomes in CKD populations. As CKD is a state of progressive vitamin K depletion and VC, research has focused on clinical trials aiming to investigate the possible beneficial effects of vitamin K in CKD and dialysis patients. In this study, we aim to review the current evidence regarding vitamin K supplementation in uremic patients.

## 1. Introduction

Cardiovascular (CV) disease is highly prevalent among Chronic Kidney Disease (CKD) patients and remains the major cause of early morbidity and mortality sustained by this population. Pre-dialysis CKD patients are at a much greater risk of experiencing a fatal CV event than of progression to end-stage kidney disease (ESKD) requiring dialysis [1] and in ESKD the CV risk is further exacerbated [2].

This increased CV risk in ESKD patients cannot be solely attributed to traditional risk factors such as hypertension, diabetes, and dyslipidemia. Additionally, these patients are overexposed to novel uremia-specific factors including oxidative stress [3,4], inflammation, disorders of mineral metabolism, and endothelial dysfunction [5]. Furthermore, calcification of arteries and cardiac valves is very common in ESKD, triggering the onset and development of arterio- or atherosclerosis and subsequent CV disease. Therefore, the increased CV death rates of ESKD patients may partially reflect the accelerated rate of arterial calcification observed in these patients.

## 2. Vascular Calcification in Chronic Kidney Disease

Calcification of the arteries starts early in life and gradually increases with age; it is a common condition in the healthy aged. The presence and degree of vascular calcification (VC) are independent and each is a strong predictor of CV morbidity and mortality. As deposition and accumulation of calcium and hydroxyapatite in any artery of the human body increases the risk of developing CV disease by 3.5 times and CV death by 3.9 times [6], it has been suggested that our biological age is partially determined by the health status of our arteries [7]. There are four distinct histopathologic patterns of arterial calcification: calcification of the intima, calcification of the media, calcification of cardiac valves, and calciphylaxis. VC can exist in any one of these forms or in combinations of them. In CKD, all these patterns might occur either alone or in combination, and the degree of VC progressively increases along with disease deterioration to ESKD [8,9]. Compared to the general population, the prevalence of arterial microcalcification is 45 times greater in CKD patients [10]. Even in early stages of CKD, calcification of the media or intima is present in 50–90% of all cases [11] and the prevalence and degree of VC are more increased in ESKD. Eight out of ten HD patients present with VC, which is tightly correlated with the duration of dialysis [12]; every year on dialysis confers a 15% increased risk of developing calcification of the coronary arteries [13]. However, although the deleterious effects of VC in CKD and ESKD patients have been known for a long time the pathophysiology of this process was not fully elucidated until recently. For more than a century, arterial calcification was believed to be a passive, progressive and untreatable process of calcium accumulation in the arterial walls. However, three decades ago this perspective changed significantly and it became evident that the calcification of arteries is not a degenerative but rather an active process starting with the osteoblastic differentiation of vascular smooth muscle cells (VSMCs) [14], a process similar to bone formation. Moreover, it became clear that the onset and development of VC is regulated by various molecules normally involved in the regulation of bone metabolism, which can act as either promoters or inhibitors of arterial calcification. Therefore, VC is the result of the disruption of balance between inhibitors and promoters in favor of the latter. In advanced CKD, the consequences of kidney dysfunction (particularly mineral dysregulation, inflammation and accumulation of uremic toxins) favor the osteogenic transition of vascular smooth muscle cells, through the activity of cytokines and enzymes such as Fibroblast Growth Factor-23 [15,16], osteocalcin [17,18,19], sclerostin [20,21,22], bone-morphogenetic proteins [16,23,24,25], osteoprotegerin [22,26,27,28], RUNX2 [16] and calcium-sensing receptor [29,30] that trigger the osteoblastic differentiation of VSMCs, thus promoting the onset and development of VC [31,32,33,34]. In addition, accumulation of uremic toxins and enhanced oxidative stress and inflammation suppress the concentration and expression of calcification inhibitors, such as Klotho [15,16,35] and pyrophosphates [36,37,38]. Although VC is highly prevalent in ESKD patients, 15% of HD patients do not exhibit calcification of the vasculature even years after initiation of dialysis [39] due to the protection of natural calcification inhibitors. Therefore, scientific research is focused on the pathophysiology of calcification inhibitors in CKD, especially their activation pathways.

## 3. Matrix Gla Protein as a Calcification Inhibitor

Matrix Gla Protein (MGP) is a 12 kDa protein containing 84 amino-acids, four or five glutamate (Glu) and three serine residues which is secreted by VSMCs in the arterial wall and expressed in the bones, heart, kidneys, cartilage and arteries. MGP was the first identified inhibitor of VC in experimental studies and has the ability to prevent as well as to reverse calcification of the vessel walls [40,41,42] through several pathways. MGP binds directly to free circulating calcium, phosphorus, and hydroxyapatite crystals and forms neutralized, inactive complexes that lose their ability to accumulate within the arterial walls [36]. MGP additionally activates the phagocytosis of these complexes by arterial macrophages and abrogates the expression of the calcification bone morphogenetic protein -2 (BMP-2) by antagonizing the binding of BMP-2 to its receptor [43,44,45,46,47]. Finally, MGP has the ability to remove calcium and extracellular matrix from the vessel walls [48]. The function and clinical importance of this inhibitor of VC was first recognized by Luo et al., who found that MGP-deficient animals (knockout models, MGP^--/--^), develop to term but die within eight weeks due to accelerated VC leading to blood vessel rupture in the aorta [41]. Moreover, data from genetic studies suggest that mutations and polymorphisms of the MGP gene lead to non-functional or partially-functional MGP, respectively, and are associated with ectopic calcification of the cartilage and the arteries as well as with CV mortality in CKD [49,50]. Therefore, a genetic basis underlying the pathogenesis of arterial calcification in CKD through de-activation of MGP might occur in CKD.

MGP belongs to the family of vitamin K-dependent proteins (VKDPs), a group of seventeen proteins involved in the regulation of bone metabolism, blood coagulation, and arterial calcification. All VKDPs have inactive Glu residues that undergo γ-carboxylation to γ-carboxyglutamate (Gla), a process requiring vitamin K as a co-factor. The carboxylation of Glu causes significant alterations in the molecular structure of VKDPs, leading to their activation. In contrast to the other VKDPs, after γ-carboxylation ΜGP needs to undergo another activation step, phosphorylation of its serine residues, which again requires vitamin K as a co-factor. Therefore, while the fully inactive form of MGP is the dephosphorylated, uncarboxylated MGP (dp-ucMGP) MGP exists in the partially inactive forms of dephosphorylated carboxylated MGP (dp-cMGP) and phosphorylated uncarboxylated MGP (p-ucMGP) as well. After carboxylation and phosphorylation, MGP is fully activated and can act as a powerful calcification inhibitor via the aforementioned molecular pathways. On the other hand, dp-ucMGP cannot bind to calcium/phosphate particles, hydroxyapatite, or BMP-2 and therefore loses the ability to act as an inhibitor of VC [51,52,53,54]. Because vitamin K is an essential co-factor for both activation steps of MGP, in vitamin K deficiency MGP is deactivated and the circulating levels of dp-ucMGP are further increased. Accumulated data from in vitro and in vivo studies has coherently detected local concentrations of dp-ucMGP around calcification sites such as atheromatic lesions and plaques, and circulating levels of dp-ucMGP are strongly correlated with the degree of VC [55,56]. Moreover, in experimental models, vitamin K deficiency (achieved by the use of the vitamin K antagonist warfarin) was accompanied by an abrupt increase in dp-ucMGP and accelerated calcification; after supplementation with high doses of vitamin K, however, MGP was activated, dp-ucMGP levels were suppressed, and calcification status was reduced by 37% [42]. The importance of this study was that it showed for the first time that supplementation with vitamin K might prevent or even reverse VC.

## 4. Vitamin K Deficiency in CKD as a Predictor of Calcification and Adverse Events

By activating VKDPs, vitamin K (a group of fat-soluble vitamins) is implicated in the regulation of bone metabolism, blood coagulation, and vascular health. Vitamin K exists in three vitamers, phylloquinone (or K1), menaquinone (K2), and menadione (K3); there are several sub-forms of K2 depending on the number of isoprenyl units and the length of the side-chain. Among these, the K2 subtype with the longest side chain, the longest half-life, and the optimal bioavailability in humans is menaquinone-7 (MK-7). For this reason, MK-7 is considered to be the optimal form of vitamin K for supplementation, and is commercially used for supplementation [57]. However, all vitamers and sub-forms of vitamin K can act as co-factors for the activation of MGP.

### 4.1. Association between Vitamin K Status and Arterial Calcification/Stiffness in CKD

Epidemiological data in the general population (NHANES II [58]) as well as ERGO [59], the Danish Diet, Cancer and Health Study [60], EPIC [61], and the PREVEND study [62] have all suggested that poor vitamin K status is independently associated with VC, mortality, and CV disease. Subclinical vitamin K depletion is highly prevalent in ESKD patients and has been associated with VC, vascular stiffness, mortality, and CV disease. The increased prevalence of vitamin K depletion in ESKD patients can be due to any of several different reasons. In this stage, ESKD patients are given strict dietary recommendations that include restriction of dairy products rich in phosphate (and vitamin K2) and green vegetables rich in potassium (and vitamin K1) [17]. The uremic environment can directly decrease the activity of vitamin K-recycling molecules and enzymes [63]; finally, certain drug agents that are commonly used by ESKD patients, such as phosphate binders, can further exacerbate vitamin K deficiency [64]. Because CKD patients suffer from accelerated calcification, several researchers have aimed to investigate the possible association between poor vitamin K status as assessed by dp-ucMGP and VC. In pre-dialysis CKD, data from a few observational studies showed that dp-ucMGP was associated with various surrogate markers of arterial calcification or stiffness. Schurgers et al. reported that in 107 patients in CKD stages 2–5, circulating dp-ucMGP gradually increased with CKD stage and was strongly and independently correlated with aortic calcification score as assessed by spiral computed tomography [65]. Likewise, in another cross-sectional study that enrolled 83 patients in CKD stages 3 to 5, dp-ucMGP was augmented according to CKD severity [66]. Moreover, plasma dp-ucMGP was independently associated with VC as assessed by abdominal aortic calcification, and was not associated with markers of vascular stiffness such as pulse-wave velocity (PWV) and cardio-ankle vascular index. However, in 67 patients with diabetic CKD dp-ucMGP failed to correlate with carotid intima-media thickness (cIMT) [50]. The biggest study to date was conducted by Puzantian et al. [67]; in 137 patients with various degrees of kidney function, subclinical vitamin K deficiency as assessed by increased levels of dp-ucMGP was an independent predictor of carotid–femoral PWV, and there was a progressive increase in dp-ucMGP as kidney function deteriorated.

In HD populations, two independent studies showed that plasma dp-ucMGP was predictive of vitamin K status and significantly correlated with abdominal aortic calcification [68,69], whereas in a cohort of 120 maintenance HD patients, Hermans et al. found that ucMGP was associated with duration of dialysis and aortic augmentation index [70]. Similarly, Fain et al. showed that in a cohort of 37 HD patients dp-ucMGP was an independent predictor of arterial stiffness and endothelial dysfunction as assessed by PWV and flow mediated dilation, respectively, even after adjustment for duration of dialysis, gender, age, diabetes, blood pressure, and history of CV disease [71]. Moreover, in a cohort of 188 HD patients, patients with more severe degrees of VC as assessed by Adragao and total calcification score had significantly lower plasma dp-cMGP levels, whereas dp-ucMGP was not correlated with the extent of VC [72]. Fusaro et al. conducted a multicenter, cross-sectional study (VIKI) that included 387 HD patients from eighteen Italian dialysis centers; they found that HD patients with calcification of the iliac arteries exhibited significantly decreased circulating levels of vitamin K2 and MK-7, while no difference was found regarding vitamin K1 and MK-4 levels [73]. Furthermore, a close link between vitamin K deficiency and calciphylaxis has been suggested as well. In a cohort of 40 HD patients (20 presenting with calciphylaxis and 20 without) Nigwekar et al. found that every 0.1-unit decrease in cMGP levels doubled the risk of calciphylaxis [74].

In order to assess a possible association between vitamin K status and CV outcomes, Chen et al. performed a meta-analysis of 21 studies and 222,592 participants and showed that increased dietary intake of either vitamin K1 or K2 was linked with a moderately reduced risk of coronary heart disease, although not with mortality. Moreover, increased circulating dp-ucMGP (which is representative of vitamin K depletion) was predictive of both all-cause and CV mortality, while uncarboxylated osteocalcin (another VKDP implicated in bone and vascular health) was not [75].

### 4.2. Association between Vitamin K Status and Clinical Hard Endpoints in CKD and ESKD

Following the accumulating data supporting a close association between subclinical vitamin K deficiency and arterial calcification/stiffness in the CKD population, several investigators have aimed to assess whether poor vitamin K status is predictive of adverse events, including mortality, CV disease, and deterioration of kidney function. Schurgers et al. were the first to show that dp-ucMGP plasma levels were predictive of all-cause mortality in a cohort of 107 pre-dialysis CKD patients [65]. Similarly, in a group of 66 patients with diabetic CKD circulating dp-ucMGP was associated with CV morbidity, mortality, and deterioration of kidney function [50,76]. In agreement with these results, Kayzer et al., enrolled 518 stable kidney transplant recipients, followed them for a median period of 9.8 years, and found that increased plasma dp-ucMGP was an independent predictor of all-cause mortality and transplant failure [77]. Finally, in a study by Schlieper et al. dp-ucMGP was an independent predictor of mortality in 188 maintenance HD patients [72].

### 4.3. The Effect of Exogenous Vitamin K Supplementation on MGP Forms in CKD and ESKD

As it has been shown in experimental animal uremic models that supplementation with vitamin K could potentially reverse arterial calcification through activation of MGP, an attractive hypothesize is that exogenous supplementation with vitamin K might ameliorate CV disease in CKD patients through activation of MGP. While the daily recommended amount of vitamin K for healthy subjects is 90–120 μg [78], the exact dosage of vitamin K intake for activation of MGP in populations with vitamin K deficiency, such as CKD patients, has not yet been defined. In a study by Schlieper et al., the authors reported that in 17 HD patients, daily supplementation with 135 μg of MK-7 for 1.5 months caused a modest 27% reduction in dp-ucMGP levels [72]. Following these results, two randomized, interventional, non-placebo-controlled, dose-finding studies were conducted in dialysis patients. Westenfeld et al. randomly divided 53 maintenance HD patients into daily intake groups of 45, 135 or 360 μg MK-7 for 1.5 month, and found that dp-ucMGP was time- and dose-dependently reduced in all three groups (a 17.9%, 36.7% and 61.1% reduction in the 45, 135 and 360 groups, respectively) [79]. Similarly, Caluwe et al. enrolled a larger sample size of 200 HD patients and randomly divided them to three parallel groups receiving 360, 720 and 1080 μg of MK-7 thrice weekly (which translates roughly to 154, 308 or 464 μg/day) for two months, and found that dp-ucMGP was decreased by 17%, 33% and 46%, respectively, in the three groups [80]. These two studies showed that even 460 μg of vitamin K2 per day failed to restore vitamin K status (as reflected by the reduction rate of dp-ucMGP) in dialysis patients, and provided the rationale for designing randomized controlled trials (RCTs) in dialysis patients. Moreover, the response rates were better in the study by Westenfeld et al., which was attributed to the fact that the patients enrolled by Caluwe et al. were much older (64.6 versus 70.8 years of mean age). In agreement with these results, a pre- and post-intervention clinical study in 50 maintenance HD patients showed that daily supplementation with 360 μg/day of MK-7 for one month was accompanied by a significant 86% decrease in circulating dp-ucMGP, with type 2 diabetes patients exhibiting the lowest response rates [69]. Therefore, as it is well established that VC is highly prominent in diabetics and the elderly these studies suggest that the effect of vitamin K intake on MGP is highly dependent on the dose, the duration of supplementation, and the population.

## 5. Interventional Studies of Vitamin K Supplementation on Arterial Calcification and Stiffness in CKD

### 5.1. Completed Studies

There is accumulating evidence suggesting a close association between vitamin K deficiency and CV disease. Following the encouraging results of animal studies, research has focused on clinical interventional studies examining the possible beneficial effects of vitamin K supplementation on CV outcomes. A meta-analysis by Lees et al. included thirteen RCTs involving 2162 patients and fourteen longitudinal studies involving 10,726 patients, and showed that exogenous supplementation with vitamin K caused a significant 9.1% reduction in the degree of VC, attributed to a 44.7% decrease in plasma dp-ucMGP concentration [81].

Seven interventional clinical studies have published results on CKD and ESKD populations thus far: two in predialysis CKD patients, two in kidney transplant recipients, and three in dialysis patients (two only in HD, and one in a mixed dialysis population of both PD and HD patients). Table 1 summarizes the interventional clinical studies of vitamin K supplementation on arterial calcification and stiffness in CKD and ESKD populations. The Vitamin K2 Supplementation and Arterial Stiffness in the Renal Transplant Population (KING) trial [82] was a single-arm, single-center, interventional clinical trial in 60 stable kidney transplant recipients. At baseline, 53% of the recruited patients presented with subclinical vitamin K deficiency and exogenous supplementation of MK-7 (360 μg/day for two months), which was accompanied by a 14.2% improvement in vascular stiffness as assessed by the change in carotid-femoral PWV. The authors found that this beneficial effect on PWV was independently associated with a 55% decrease in plasma dp-ucMGP levels. Despite the short period of treatment, this study produced positive results; however, the outcome was the change in a surrogate marker of arterial stiffness, not a hard clinical endpoint. The second study in this population, the Vitamin K for kidney transplant organ recipients: investigating vessel stiffness (VIKTORIES) study [83], was a single-center parallel group RCT in which 90 kidney transplant recipients were randomized to 5 mg of menadiol diphosphate (a synthetic form of vitamin K) or a placebo thrice weekly for one year; the outcomes were change in arterial stiffness as assessed by aortic distensibility from magnetic resonance imaging and VC as assessed by computer tomography-based coronary calcification score. After the treatment period, the authors found no effect on either VC or vascular stiffness between the groups. Although the 90 patients were initially divided into groups, only 72 completed the study. Moreover, this was the first study to evaluate exogenous supplementation of menadiol diphosphate in this population, and there were no previous dose-finding studies regarding the exact amount of it needed to activate MGP. A third limitation of this study was that there were constraints on the measurement of dp-ucMGP; the authors reported that they could measure only absolute values of dp-ucMGP above 900 pmol/L, whereas values below 900 could not be reliably quantified and were therefore considered to be equal to 900 pmol/L; thus, patients with severe vitamin K deficiency could not be identified. In the 72 patients who completed the study, the authors reported that compared to the placebo group the active group presented a modest improvement in vitamin K status, with a mean difference in dp-ucMGP of −186 versus −12 pmol/L in the active and placebo group, respectively. Because the mean circulating dp-ucMGP in the vitamin K group was 1173 pmol/L, it became evident that supplementation with vitamin K did not fully restore vitamin K status, and that the dosage might therefore be considered undertherapeutic. Finally, at baseline the vitamin K group had increased proteinuria compared to the placebo group (12 versus < 3 mg/mmol) and higher prevalence of both diabetes (28.9% versus 15.6%) and CV disease (peripheral arterial disease was present at 6.7% versus 0%, heart failure was present at 6.7% versus 0%, ischemic heart disease at 37.8% versus 26.7%, and ischemic attack or stroke was 4.4% versus 2.2%), whereas all participants at baseline presented extended VC. Bearing in mind that diabetics and patients with heavy VC or established CV disease might have a low response to treatment with vitamin K, the results of this study might be due to this issue.

In pre-dialysis CKD populations two studies have reported results to date. Kurnatowska et al. [84], randomized 42 CKD patients in stages 3–5 with a background history of VC to either 10 μg/day of vitamin D or combination of vitamin D and K (10 μg/day vitamin D plus 90 μg/day of MK-7) for nine months and found that compared to Vitamin D alone a combination of D and K resulted in decreased progression of cIMT (a surrogate marker of VC) and significantly reduced circulating dp-ucMGP. Moreover, the K and D group presented a non-statistically significant improvement in coronary calcification score, which was even more pronounced when the authors removed from the statistical analysis four patients with markedly increased calcification scores. This was one of the first interventional studies in this research area to highlight the synergy and interaction between vitamins K and D; however, the small sample size, the short study period, and the low MK-7 dosage should be acknowledged as limitations. The Vitamin K therapy to improve vascular health in patients with chronic kidney disease (K4Kidneys) RCT showed that in 159 CKD patients in stages 3b-4 daily intake of 400 μg MK-7 for one year resulted in modest reduction of dp-ucMGP and improved insulin resistance, although it had no effect on arterial calcification or stiffness as assessed by PWV and abdominal calcification score [85]. The mean age of enrolled patients was lower than that of typical CKD 3b-4 patients, and it could be hypothesized that the study population did not exhibit severe vitamin K depletion.

Oikonomaki et al. [86], performed a study with an open-label placebo-controlled design in a population of 102 maintenance HD patients, and found that daily intake of 200 μg MK-7 for one year decreased circulating ucMGP levels by 46% while having no effect on the progression of VC as assessed by Agatston score of the abdominal aorta. In this study, ucMGP (and not dp-ucMGP) was assessed as a marker of vitamin K depletion, and the dosage of MK-7 was relatively low; which is probably why ucMGP was only reduced by 46% in the active group. Moreover, the study had a high drop-out rate of 51%, and only 52 patients were ultimately included in the analysis. Similarly, the Effect of Vitamin K2 (MK7) on Cardiovascular and Bone Disease in Dialysis Patients (RenaKvit) RCT [87] recently reported that daily treatment with 360 μg MK-7 for two years in 21 ESKD patients undergoing either HD or PD failed to show any beneficial effect on aortic stiffness or calcification. The main drawbacks of this study were its small sample size and high drop-out rate; of 641 patients originally assessed, 48 only were enrolled and only 21 ultimately completed the trial. Moreover, the population was heterogenous regarding dialysis modality, and circulating dp-ucMGP decreased by only 40% in the vitamin K group. Finally, roughly nine of ten patients in the K group were treated with phosphate binders such as sevelamer, which have been reported to limit the biologic availability of vitamin K [64]. The Effect on Vascular Calcification of Replacing Warfarin by Rivaroxaban With or Without Vitamin K2 in Hemodialysis Patients (Valkyrie) RCT [88] enrolled 132 HD patients with atrial fibrillation and randomized them to a vitamin K antagonist (warfarin), rivaroxaban, or rivaroxaban combined with 2000 μg of MK-7 thrice weekly. After 1.5 years of treatment, MK-7 failed to show any beneficial effect on various markers of VC and stiffness, including PWV, coronary artery calcification score, and calcification of the cardiac valves. Although the reduction in dp-ucMGP was significantly higher in the rivaroxaban group and even higher in the rivaroxaban plus K group, at the end of the study circulating dp-ucMGP levels in this group remained high. It should be noted that the mean age of the patients included in the K2 group was 79.6 years, and therefore the excessive calcification of arteries at this age may not be amenable to any reversal strategy.

### 5.2. Ongoing Studies That Hαve Not Yet Published Results 

To date, data regarding the effect of exogenous supplementation of vitamin K in CKD/ESKD patients remain limited. However, we expect that the results of several intensive ongoing trials will shed light on this area of research. Currently-ongoing interventional RCTs regarding vitamin K supplementation in these populations include the Inhibit Progression of Coronary Artery Calcification with Vitamin K1 in Hemodialysis participants (iPACK-HD) [89], Treatment to Reduce Vascular Calcification in Hemodialysis Patients Using Vitamin K (TReVasc-HDK) [90], Vitamin K1 to Slow Progression of Vascular Calcification in HD Patients (VitaVasK) [91], Vitamin K to Slow Progression of Cardiovascular Disease Risk in Hemodialysis Patients (Vita-K ‘n’ CKD, registered on clinicaltrials.gov, reference number NCT03311321), Universidad Católica de Salta-Vitamin K2 Supplementation and Vascular Calcification (UCASAL-VITK, registered on clinicaltrials.gov with reference number NCT04539418 and the first to assess intravenous supplementation of MK-7 in HD patients), and the Vitamin K in Peritoneal Dialysis (VIKIPEDIA) study, the first to assess MK-7 supplementation in PD (registered on clinicaltrials.gov, reference number NCT04900610).

## 6. Questions and Areas of Debate Regarding Vitamin K Intake in CKD/ESRD

### 6.1. What Dosage Is Effective for Cardiovascular Protection?

To date, the optimal daily amount of vitamin K for ESKD patients has not yet been determined [92]. However, even 460 μg per day of vitamin K2 intake failed to normalize dp-ucMGP levels in maintenance dialysis patients, and might therefore be considered under-therapeutic. Although the majority of published or ongoing trials in CKD or ESKD population have adopted a daily dosage of 200–500 μg of vitamin K2, two new RCTs registered on clinicaltrials.gov, the VIKIPEDIA and the UCASAL-VITK studies, propose higher daily dosages of vitamin K. The VIKIPEDIA study will assess the effect of 1000 μg/day of MK-7 on arterial stiffness and CV disease in PD patients, whereas the UCASAL-VITK link will investigate whether intravenous supplementation with MK-7 2000 μg thrice weekly can improve markers of VC in a cohort of maintenance HD patients.

Suboptimal vitamin K and D levels have been associated with impaired bone health and increased CV risk [92]. It has become evident that there is a close interaction and joint association between these two vitamins and vascular outcomes [93,94]. Vitamin D promotes the production of MGP and other VKDPs, whereas vitamin K and D work together to maintain calcium homeostasis in bones and circulation [93,95]. Although maintaining optimal levels of vitamin D is highly important for the function of vitamin K, this issue has been repeatedly overlooked or neglected in several interventional studies examining the effect of vitamin K in CKD/ESKD patients. A small RCT in 42 predialysis CKD patients showed that when compared to vitamin D alone, a combination of vitamin K2 and D resulted in favorable results regarding dp-ucMGP reduction but had no impact on progression of VC [84]. Moreover, a very recent randomized interventional non-placebo, controlled trial was performed in 60 ESKD children undergoing maintenance HD, who were randomized to receive 100 μg of MK-7 or 10 μg of native vitamin D, a combination of both, or standard care (no vitamins) every day for four months; the results showed that when compared to the other groups those who received the combination of both vitamins presented the highest reduction in dp-ucMGP [96]. Therefore, in RCTs investigating the effect of vitamin K on CKD patients vitamin D status should be always assessed and corrected at baseline [97].

### 6.2. Methods for Measuring Vitamin K Status

Thus far, various methods for the quantification of vitamin K status have been used in clinical studies. The first epidemiological studies used food questionnaires to assess daily intake of vitamin K; however, this method has certain limitations. In other studies, circulating K1 in serum or plasma was assessed; however, levels of K1 are highly dependent on lipids and lipoproteins, and do not reflect the levels of K2. It has become evident that an optimal assessment method should quantify both vitamin K status and the availability of the vitamin that can be used for activation of VKDPs such as MGP. In addition to circulating dp-ucMGP, Proteins Induced by Vitamin K Absence (PIVKA-II), a marker that collectively includes all the inactive under-carboxylated forms of VKDPs, is considered an optimal marker of subclinical vitamin K deficiency [98]. Among these two markers, PIVKA-II represents the bioavailability of vitamin K for carboxylation only, while dp-ucMGP reflects the bioactivity of vitamin K for both carboxylation and phosphorylation over a span of weeks [99].

### 6.3. Is Vitamin K Safe and Well-Tolerated?

The interventional clinical studies performed to date coherently show that vitamin K is safe and well-tolerated in CKD, kidney transplant recipients, and dialysis patients. The adverse events that have been recorded are very mild and mostly include gastrointestinal symptoms, and toxicity has never been reported. A systematic review of nine clinical trials [100] highlighted that vitamin K is safe to administer; no serious adverse events or death were reported with vitamin K intake. Moreover, in the Valkyrie trial [90] the incidence of stroke did not differ between the groups receiving rivaroxaban and rivaroxaban plus MK-7, and hemorrhagic stroke occurred only in the warfarin group. It should be noted that toxicity from vitamin K has never been reported in experimental studies, even with daily intake of 20 mg of vitamin K1 in pregnancy [101] or 2000 mg/kg MK-7 by body weight [102].

### 6.4. Should CKD Patients Be Advised to Receive Vitamin K?

Recent interventional studies examining vitamin K supplementation for VC in CKD and ESKD have failed to show any clear-cut benefit, thus suggesting that the limited dietary intake of vitamin K cannot be the only mechanism responsible for vitamin K depletion in these patients. In a very recent study, Kaesler et al. [103] orally administered a combination of vitamin K1 and K2 (MK-4 and MK-7) in ten HD patients and nine healthy controls and found that, when compared to healthy controls, HD patients exhibited a significantly increased uptake and incorporation of MK-4 into high (HDL) and low density lipoproteins (LDL). Moreover, in this study, in vitro, HDL particles isolated from HD patients presented very low (almost absent) MK-7 incorporation and when HDL particles from the study cohort were spiked with MK-7, only those isolated from healthy controls (but not those from HD patients) presented significantly decreased ucMGP levels in VSMCs. In the same study, the authors analyzed data from a cohort of 1051 type 2 diabetic mellitus ESKD patients undergoing maintenance HD and found that patients receiving a combination of atorvastatin with a proton pump inhibitor had the highest circulating levels of PIVKA-II, an established marker of poor vitamin K status. This study expands our understanding, provides novel insights into vitamin K metabolism in uremia, and suggests that altered uptake and transportation of vitamin K by lipoproteins in advanced CKD might be another mechanism (besides reduced vitamin K intake) underlying the poor vitamin K status of HD patients. This crucial alteration in the pharmacology of MK-7 in uremia might partially explain the negative RCTs regarding MK-7 supplementation in predialysis CKD patients.

Moreover, there are numerous knowledge gaps and research questions that have remained unanswered to date regarding the supplementation of vitamin K in these populations, including the best form, dosage, duration of treatment, method of measuring vitamin K status, and expected response to treatment. However, it has been coherently shown that vitamin K is safe, well-tolerated and poses no danger of major adverse events or toxicity. Therefore, the first rule when introducing a novel therapeutic agent, “First, do no harm”, is probably respected; whether the “do good” value is supported remains to be elucidated based on the results of current ongoing clinical trials.

## 7. Conclusions

In summation, although increased vitamin K intake is coherently associated with decreased risk of VC and CV disease in the general population, there is no clear-cut evidence favoring vitamin K supplementation for CKD and ESKD at present.

In CKD, vitamin K deficiency is highly prevalent, starts early, progresses along with disease progression to ESKD, and increases the risk of accelerated VC and CV morbidity and mortality. Currently, there is keen scientific interest on the potential clinical role of vitamin K in the pathogenesis and development of arterial calcification and stiffness in CKD and ESKD. In these populations, dp-ucMGP reflects poor vitamin K status and is significantly reduced with vitamin K2 (especially MK-7) supplementation. However, thus far the results of such supplementation on the progression of VC remain ambiguous and contraindicatory. There is a need for large and well-designed RCTs evaluating the effect of vitamin K supplementation on CKD and ESKD in order to permit the drawing of more definite conclusions.

## Figures and Tables

**Table 1:** Interventional studies of the effects of Vitamin K supplementation on arterial calcification and stiffness in CKD and ESKD.

Clinical Trials							
	KING	VIKTORIES	KURNATOWSKA	K4KIDNEYS	OIKONOMAKI	RENAKVIT	VALKYRIE
**Reference**	[82]	[83]	[84]	[85]	[86]	[87]	[88]
**Year**	2017	2021	2015	2020	2019	2021	2020
**Patients**	Kidney transplant recipients	Kidney transplant recipients	CKD, stages 3–5	CKD, predialysis	HD	HD/PD	HD with AF
**N**	60	90	42	159	52	21	132
**Vitamin K**	MK-7	Menadiol diphosphate	MK-7	MK-7	MK-7	MK-7	MK-7
**Dose**	360 μg/day	5 mg×3/week	90 μg/day	400 μg/day	200 μg/day	360 μg/day	200 μg × 3/week
**Duration**	60 days	360 days	270 days	360 days	360 days	720 days	540 days
**Groups**	Single group	K group/placebo	10 μg/day D/D + K	K group/placebo	Single group	K group/placebo	Warfarin/ Rivaroxaban/ Rivaroxaban + K
**Outcome**	14.2% reduction of PWV	No effect on VC or vascular stiffness	Reduction of CIMT progression Trend towards CACS improvement	No effect on PWV or VC	No effect on Agatston score	No effect on VC or vascular stiffness	No effect on VC, vascular stiffness or cardiac valve calcification

AF, Atrial Fibrillation; CACS, coronary calcification score; CKD, chronic kidney disease; cIMT, carotid intima-media thickness; HD, hemodialysis; MK-7, menaquinone 7; PD, Peritoneal Dialysis; PWV, pulse-wave velocity; VC, Vascular Calcification.

## Data Availability

Not applicable.

## References

[1] Foley R.N., Murray A.M., Li S., Herzog C.A., McBean A.M., Eggers P.W., Collins A.J. (2005). Chronic kidney disease and the risk for cardiovascular disease, renal replacement, and death in the United States Medicare population, 1998 to 1999. J. Am. Soc. Nephrol..

[2] Levey A.S., Beto J.A., Coronado B.E., Eknoyan G., Foley R.N., Kasiske B.L., Klag M.J., Mailloux L.U., Manske C.L., Meyer K.B. (1998). Controlling the epidemic of cardiovascular disease in chronic renal disease: What do we know? What do we need to learn? Where do we go from here? National Kidney Foundation Task Force on Cardiovascular Disease. Am. J. Kidney Dis. Off. J. Natl. Kidney Found..

[3] Liakopoulos V., Roumeliotis S., Gorny X., Dounousi E., Mertens P.R. (2017). Oxidative Stress in Hemodialysis Patients: A Review of the Literature. Oxid. Med. Cell. Longev..

[4] Liakopoulos V., Roumeliotis S., Gorny X., Eleftheriadis T., Mertens P.R. (2017). Oxidative Stress in Patients Undergoing Peritoneal Dialysis: A Current Review of the Literature. Oxid. Med. Cell. Longev..

[5] Roumeliotis S., Mallamaci F., Zoccali C. (2020). Endothelial Dysfunction in Chronic Kidney Disease, from Biology to Clinical Outcomes: A 2020 Update. J. Clin. Med..

[6] Rennenberg R., Kessels A., Schurgers L., Van Engelshoven J., De Leeuw P., Kroon A. (2009). Vascular calcifications as a marker of increased cardiovascular risk: A meta-analysis. Vasc. Health Risk Manag..

[7] Raggi P., Shaw L.J., Berman D.S., Callister T.Q. (2004). Prognostic value of coronary artery calcium screening in subjects with and without diabetes. J. Am. Coll. Cardiol..

[8] Roumeliotis A., Roumeliotis S., Panagoutsos S., Theodoridis M., Argyriou C., Tavridou A., Georgiadis G.S. (2019). Carotid intima-media thickness is an independent predictor of all-cause mortality and cardiovascular morbidity in patients with diabetes mellitus type 2 and chronic kidney disease. Ren. Fail..

[9] Temmar M., Liabeuf S., Renard C., Czernichow S., Esper N.E., Shahapuni I., Presne C., Makdassi R., Andrejak M., Tribouilloy C. (2010). Pulse wave velocity and vascular calcification at different stages of chronic kidney disease. J. Hypertens..

[10] O’neill W.C., Lomashvili K.A. (2010). Recent progress in the treatment of vascular calcification. Kidney Int..

[11] Porter C.J., Stavroulopoulos A., Roe S.D., Pointon K., Cassidy M.J. (2007). Detection of coronary and peripheral artery calcification in patients with chronic kidney disease stages 3 and 4, with and without diabetes. Nephrol. Dial. Transplant..

[12] Raggi P., Boulay A., Chasan-Taber S., Amin N., Dillon M., Burke S.K., Chertow G.M. (2002). Cardiac calcification in adult hemodialysis patients: A link between end-stage renal disease and cardiovascular disease?. J. Am. Coll. Cardiol..

[13] Yuen D., Pierratos A., Richardson R.M., Chan C.T. (2006). The natural history of coronary calcification progression in a cohort of nocturnal haemodialysis patients. Nephrol. Dial. Transplant..

[14] Roumeliotis S., Roumeliotis A., Dounousi E., Eleftheriadis T., Liakopoulos V. (2020). Biomarkers of vascular calcification in serum. Adv. Clin. Chem..

[15] Lu X., Hu M.C. (2017). Klotho/FGF23 Axis in Chronic Kidney Disease and Cardiovascular Disease. Kidney Dis..

[16] Hortells L., Sosa C., Guillen N., Lucea S., Millan A., Sorribas V. (2017). Identifying early pathogenic events during vascular calcification in uremic rats. Kidney Int..

[17] Cranenburg E.C., Schurgers L.J., Uiterwijk H.H., Beulens J.W., Dalmeijer G.W., Westerhuis R., Magdeleyns E.J., Herfs M., Vermeer C., Laverman G.D. (2012). Vitamin K intake and status are low in hemodialysis patients. Kidney Int..

[18] Nagata Y., Inaba M., Imanishi Y., Okazaki H., Yamada S., Mori K., Shoji S., Koyama H., Okuno S. (2015). Increased undercarboxylated osteocalcin/intact osteocalcin ratio in patients undergoing hemodialysis. Osteoporos. Int..

[19] Pilkey R.M., Morton A.R., Boffa M.B., Noordhof C., Day A.G., Su Y., Miller L.M., Koschinsky M.L., Booth S.L. (2007). Subclinical vitamin K deficiency in hemodialysis patients. Am. J. Kidney Dis..

[20] Zeng C., Guo C., Cai J., Tang C., Dong Z. (2018). Serum sclerostin in vascular calcification and clinical outcome in chronic kidney disease. Diab. Vasc. Dis. Res..

[21] Brandenburg V.M., D’Haese P., Deck A., Mekahli D., Meijers B., Neven E., Evenepoel P. (2016). From skeletal to cardiovascular disease in 12 steps-the evolution of sclerostin as a major player in CKD-MBD. Pediatr. Nephrol..

[22] Morena M., Jaussent I., Dupuy A.M., Bargnoux A.S., Kuster N., Chenine L., Leray-Moragues H., Klouche K., Vernhet H., Canaud B. (2015). Osteoprotegerin and sclerostin in chronic kidney disease prior to dialysis: Potential partners in vascular calcifications. Nephrol. Dial. Transpl..

[23] Dorai H., Vukicevic S., Sampath T.K. (2000). Bone morphogenetic protein-7 (osteogenic protein-1) inhibits smooth muscle cell proliferation and stimulates the expression of markers that are characteristic of SMC phenotype in vitro. J. Cell. Physiol..

[24] Li T., Surendran K., Zawaideh M.A., Mathew S., Hruska K.A. (2004). Bone morphogenetic protein 7: A novel treatment for chronic renal and bone disease. Curr. Opin. Nephrol. Hypertens..

[25] Rong S., Zhao X., Jin X., Zhang Z., Chen L., Zhu Y., Yuan W. (2014). Vascular calcification in chronic kidney disease is induced by bone morphogenetic protein-2 via a mechanism involving the Wnt/beta-catenin pathway. Cell. Physiol. Biochem..

[26] Nitta K., Akiba T., Uchida K., Kawashima A., Yumura W., Kabaya T., Nihei H. (2003). The progression of vascular calcification and serum osteoprotegerin levels in patients on long-term hemodialysis. Am. J. Kidney Dis..

[27] Morena M., Jaussent I., Halkovich A., Dupuy A.M., Bargnoux A.S., Chenine L., Leray-Moragues H., Klouche K., Vernhet H., Canaud B. (2012). Bone biomarkers help grading severity of coronary calcifications in non dialysis chronic kidney disease patients. PLoS ONE.

[28] Morena M., Terrier N., Jaussent I., Leray-Moragues H., Chalabi L., Rivory J.-P., Maurice F., Delcourt C., Cristol J.-P., Canaud B. (2006). Plasma osteoprotegerin is associated with mortality in hemodialysis patients. J. Am. Soc. Nephrol..

[29] Eren P.A., Turan K., Berber I., Canbakan M., Kara M., Tellioglu G., Bugan U., Sevinc C., Turkmen F., Titiz M.I. (2009). The clinical significance of parathyroid tissue calcium sensing receptor gene polymorphisms and expression levels in end-stage renal disease patients. Clin. Nephrol..

[30] Massy Z.A., Henaut L., Larsson T.E., Vervloet M.G. (2014). Calcium-sensing receptor activation in chronic kidney disease: Effects beyond parathyroid hormone control. Semin. Nephrol..

[31] Moe S.M., Reslerova M., Ketteler M., O’Neill K., Duan D., Koczman J., Westenfeld R., Jahnen-Dechent W., Chen N.X. (2005). Role of calcification inhibitors in the pathogenesis of vascular calcification in chronic kidney disease (CKD). Kidney Int..

[32] Hujairi N.M., Afzali B., Goldsmith D.J. (2004). Cardiac calcification in renal patients: What we do and don’t know. Am. J. Kidney Dis..

[33] Schlieper G., Schurgers L., Brandenburg V., Reutelingsperger C., Floege J. (2016). Vascular calcification in chronic kidney disease: An update. Nephrol. Dial. Transpl..

[34] Schlieper G., Westenfeld R., Brandenburg V., Ketteler M. (2007). Inhibitors of calcification in blood and urine. Semin. Dial..

[35] Hu M.C., Shi M., Zhang J., Quiñones H., Griffith C., Kuro-o M., Moe O.W. (2011). Klotho deficiency causes vascular calcification in chronic kidney disease. J. Am. Soc. Nephrol..

[36] Reynolds J.L., Joannides A.J., Skepper J.N., McNair R., Schurgers L.J., Proudfoot D., Jahnen-Dechent W., Weissberg P.L., Shanahan C.M. (2004). Human vascular smooth muscle cells undergo vesicle-mediated calcification in response to changes in extracellular calcium and phosphate concentrations: A potential mechanism for accelerated vascular calcification in ESRD. J. Am. Soc. Nephrol..

[37] Lomashvili K.A., Garg P., Narisawa S., Millan J.L., O’Neill W.C. (2008). Upregulation of alkaline phosphatase and pyrophosphate hydrolysis: Potential mechanism for uremic vascular calcification. Kidney Int..

[38] Mathew S., Tustison K.S., Sugatani T., Chaudhary L.R., Rifas L., Hruska K.A. (2008). The mechanism of phosphorus as a cardiovascular risk factor in CKD. J. Am. Soc. Nephrol..

[39] Moe S.M., O’Neill K.D., Reslerova M., Fineberg N., Persohn S., Meyer C.A. (2004). Natural history of vascular calcification in dialysis and transplant patients. Nephrol. Dial. Transpl..

[40] Munroe P.B., Olgunturk R.O., Fryns J.P., Van Maldergem L., Ziereisen F., Yuksel B., Gardiner R.M., Chung E. (1999). Mutations in the gene encoding the human matrix Gla protein cause Keutel syndrome. Nat. Genet..

[41] Luo G., Ducy P., McKee M.D., Pinero G.J., Loyer E., Behringer R.R., Karsenty G. (1997). Spontaneous calcification of arteries and cartilage in mice lacking matrix GLA protein. Nature.

[42] Schurgers L.J., Spronk H.M., Soute B.A., Schiffers P.M., DeMey J.G., Vermeer C. (2007). Regression of warfarin-induced medial elastocalcinosis by high intake of vitamin K in rats. Blood.

[43] Wallin R., Cain D., Hutson S.M., Sane D.C., Loeser R. (2000). Modulation of the binding of matrix Gla protein (MGP) to bone morphogenetic protein-2 (BMP-2). Thromb. Haemost..

[44] Bostrom K., Tsao D., Shen S., Wang Y., Demer L.L. (2001). Matrix GLA protein modulates differentiation induced by bone morphogenetic protein-2 in C3H10T1/2 cells. J. Biol. Chem..

[45] Sweatt A., Sane D.C., Hutson S.M., Wallin R. (2003). Matrix Gla protein (MGP) and bone morphogenetic protein-2 in aortic calcified lesions of aging rats. J. Thromb. Haemost..

[46] Zebboudj A.F., Imura M., Bostrom K. (2002). Matrix GLA protein, a regulatory protein for bone morphogenetic protein-2. J. Biol. Chem..

[47] Zebboudj A.F., Shin V., Bostrom K. (2003). Matrix GLA protein and BMP-2 regulate osteoinduction in calcifying vascular cells. J. Cell. Biochem..

[48] Hackeng T.M., Rosing J., Spronk H.M., Vermeer C. (2001). Total chemical synthesis of human matrix Gla protein. Protein Sci..

[49] Brancaccio D., Biondi M.L., Gallieni M., Turri O., Galassi A., Cecchini F., Russo D., Andreucci V., Cozzolino M. (2005). Matrix GLA protein gene polymorphisms: Clinical correlates and cardiovascular mortality in chronic kidney disease patients. Am. J. Nephrol..

[50] Roumeliotis S., Roumeliotis A., Panagoutsos S., Giannakopoulou E., Papanas N., Manolopoulos V.G., Passadakis P., Tavridou A. (2017). Matrix Gla protein T-138C polymorphism is associated with carotid intima media thickness and predicts mortality in patients with diabetic nephropathy. J. Diabetes Complicat..

[51] Schurgers L.J., Spronk H.M., Skepper J.N., Hackeng T.M., Shanahan C.M., Vermeer C., Weissberg P.L., Proudfoot D. (2007). Post-translational modifications regulate matrix Gla protein function: Importance for inhibition of vascular smooth muscle cell calcification. J. Thromb. Haemost..

[52] Schurgers L.J., Teunissen K.J., Knapen M.H., Kwaijtaal M., van Diest R., Appels A., Reutelingsperger C.P., Cleutjens J.P., Vermeer C. (2005). Novel conformation-specific antibodies against matrix gamma-carboxyglutamic acid (Gla) protein: Undercarboxylated matrix Gla protein as marker for vascular calcification. Arterioscler Thromb Vasc Biol.

[53] Murshed M., Schinke T., McKee M.D., Karsenty G. (2004). Extracellular matrix mineralization is regulated locally; different roles of two gla-containing proteins. J. Cell Biol..

[54] Shearer M.J. (1995). Vitamin K. Lancet.

[55] Roijers R.B., Debernardi N., Cleutjens J.P., Schurgers L.J., Mutsaers P.H., van der Vusse G.J. (2011). Microcalcifications in early intimal lesions of atherosclerotic human coronary arteries. Am. J. Pathol..

[56] Chatrou M.L., Cleutjens J.P., van der Vusse G.J., Roijers R.B., Mutsaers P.H., Schurgers L.J. (2015). Intra-Section Analysis of Human Coronary Arteries Reveals a Potential Role for Micro-Calcifications in Macrophage Recruitment in the Early Stage of Atherosclerosis. PLoS ONE.

[57] Card D.J., Shearer M.J., Schurgers L.J., Gomez K., Harrington D.J. (2016). What’s in a name? The pharmacy of vitamin K. Br. J. Haematol..

[58] Cheung C.L., Sahni S., Cheung B.M., Sing C.W., Wong I.C. (2015). Vitamin K intake and mortality in people with chronic kidney disease from NHANES III. Clin. Nutr..

[59] Geleijnse J.M., Vermeer C., Grobbee D.E., Schurgers L.J., Knapen M.H., van der Meer I.M., Hofman A., Witteman J.C. (2004). Dietary intake of menaquinone is associated with a reduced risk of coronary heart disease: The Rotterdam Study. J. Nutr..

[60] Bellinge J.W., Dalgaard F., Murray K., Connolly E., Blekkenhorst L.C., Bondonno C.P., Lewis J.R., Sim M., Croft K.D., Gislason G. (2021). Vitamin K Intake and atherosclerotic cardiovascular disease in the danish diet cancer and health study. J. Am. Heart Assoc..

[61] Nimptsch K., Rohrmann S., Kaaks R., Linseisen J. (2010). Dietary vitamin K intake in relation to cancer incidence and mortality: Results from the Heidelberg cohort of the European Prospective Investigation into Cancer and Nutrition (EPIC-Heidelberg). Am. J. Clin. Nutr..

[62] Riphagen I.J., Keyzer C.A., Drummen N.E.A., de Borst M.H., Beulens J.W.J., Gansevoort R.T., Geleijnse J.M., Muskiet F.A.J., Navis G., Visser S.T. (2017). Prevalence and Effects of Functional Vitamin K Insufficiency: The PREVEND Study. Nutrients.

[63] Kaesler N., Magdeleyns E., Herfs M., Schettgen T., Brandenburg V., Fliser D., Vermeer C., Floege J., Schlieper G., Kruger T. (2014). Impaired vitamin K recycling in uremia is rescued by vitamin K supplementation. Kidney Int..

[64] Neradova A., Schumacher S., Hubeek I., Lux P., Schurgers L., Vervloet M. (2017). Phosphate binders affect vitamin K concentration by undesired binding, an in vitro study. BMC Nephrol..

[65] Schurgers L.J., Barreto D.V., Barreto F.C., Liabeuf S., Renard C., Magdeleyns E.J., Vermeer C., Choukroun G., Massy Z.A. (2010). The circulating inactive form of matrix gla protein is a surrogate marker for vascular calcification in chronic kidney disease: A preliminary report. Clin. J. Am. Soc. Nephrol..

[66] Thamratnopkoon S., Susantitaphong P., Tumkosit M., Katavetin P., Tiranathanagul K., Praditpornsilpa K., Eiam-Ong S. (2017). Correlations of Plasma Desphosphorylated Uncarboxylated Matrix Gla Protein with Vascular Calcification and Vascular Stiffness in Chronic Kidney Disease. Nephron.

[67] Puzantian H., Akers S.R., Oldland G., Javaid K., Miller R., Ge Y., Ansari B., Lee J., Suri A., Hasmath Z. (2018). Circulating Dephospho-Uncarboxylated Matrix Gla-Protein Is Associated With Kidney Dysfunction and Arterial Stiffness. Am. J. Hypertens..

[68] Delanaye P., Krzesinski J.M., Warling X., Moonen M., Smelten N., Medart L., Pottel H., Cavalier E. (2014). Dephosphorylated-uncarboxylated Matrix Gla protein concentration is predictive of vitamin K status and is correlated with vascular calcification in a cohort of hemodialysis patients. BMC Nephrol..

[69] Aoun M., Makki M., Azar H., Matta H., Chelala D.N. (2017). High Dephosphorylated-Uncarboxylated MGP in Hemodialysis patients: Risk factors and response to vitamin K2, A pre-post intervention clinical trial. BMC Nephrol..

[70] Hermans M.M., Vermeer C., Kooman J.P., Brandenburg V., Ketteler M., Gladziwa U., Rensma P.L., Leunissen K.M., Schurgers L.J. (2007). Undercarboxylated matrix GLA protein levels are decreased in dialysis patients and related to parameters of calcium-phosphate metabolism and aortic augmentation index. Blood Purif..

[71] Fain M.E., Kapuku G.K., Paulson W.D., Williams C.F., Raed A., Dong Y., Knapen M.H.J., Vermeer C., Pollock N.K. (2018). Inactive Matrix Gla Protein, Arterial Stiffness, and Endothelial Function in African American Hemodialysis Patients. Am. J. Hypertens..

[72] Schlieper G., Westenfeld R., Kruger T., Cranenburg E.C., Magdeleyns E.J., Brandenburg V.M., Djuric Z., Damjanovic T., Ketteler M., Vermeer C. (2011). Circulating nonphosphorylated carboxylated matrix gla protein predicts survival in ESRD. J. Am. Soc. Nephrol..

[73] Fusaro M., Tripepi G., Plebani M., Politi C., Aghi A., Taddei F., Schileo E., Zaninotto M., Manna G.L., Cianciolo G. (2021). The vessels-bone axis: Iliac artery calcifications, vertebral fractures and vitamin K from VIKI study. Nutrients.

[74] Nigwekar S.U., Bloch D.B., Nazarian R.M., Vermeer C., Booth S.L., Xu D., Thadhani R.I., Malhotra R. (2017). Vitamin K-Dependent Carboxylation of Matrix Gla Protein Influences the Risk of Calciphylaxis. J. Am. Soc. Nephrol..

[75] Chen H.G., Sheng L.T., Zhang Y.B., Cao A.L., Lai Y.W., Kunutsor S.K., Jiang L., Pan A. (2019). Association of vitamin K with cardiovascular events and all-cause mortality: A systematic review and meta-analysis. Eur. J. Nutr..

[76] Roumeliotis S., Roumeliotis A., Stamou A., Leivaditis K., Kantartzi K., Panagoutsos S., Liakopoulos V. (2020). The Association of dp-ucMGP with Cardiovascular Morbidity and Decreased Renal Function in Diabetic Chronic Kidney Disease. Int. J. Mol. Sci..

[77] Keyzer C.A., Vermeer C., Joosten M.M., Knapen M.H., Drummen N.E., Navis G., Bakker S.J., de Borst M.H. (2015). Vitamin K status and mortality after kidney transplantation: A cohort study. Am. J. Kidney Dis..

[78] National Institutes of Health—Office of Dietary Supplements (2001). Nutrient Recommendations: Dietary Reference Intakes.

[79] Westenfeld R., Krueger T., Schlieper G., Cranenburg E.C., Magdeleyns E.J., Heidenreich S., Holzmann S., Vermeer C., Jahnen-Dechent W., Ketteler M. (2012). Effect of vitamin K2 supplementation on functional vitamin K deficiency in hemodialysis patients: A randomized trial. Am. J. Kidney Dis..

[80] Caluwe R., Vandecasteele S., Van Vlem B., Vermeer C., De Vriese A.S. (2014). Vitamin K2 supplementation in haemodialysis patients: A randomized dose-finding study. Nephrol. Dial. Transplant..

[81] Lees J.S., Chapman F.A., Witham M.D., Jardine A.G., Mark P.B. (2019). Vitamin K status, supplementation and vascular disease: A systematic review and meta-analysis. Heart.

[82] Mansour A.G., Hariri E., Daaboul Y., Korjian S., El Alam A., Protogerou A.D., Kilany H., Karam A., Stephan A., Bahous S.A. (2017). Vitamin K2 supplementation and arterial stiffness among renal transplant recipients—A single-arm, single-center clinical trial. J. Am. Soc. Hypertens..

[83] Lees J.S., Rankin A.J., Gillis K.A., Zhu L.Y., Mangion K., Rutherford E., Roditi G.H., Witham M.D., Chantler D., Panarelli M. (2021). The ViKTORIES trial: A randomized, double-blind, placebo-controlled trial of vitamin K supplementation to improve vascular health in kidney transplant recipients. Am. J. Transplant..

[84] Kurnatowska I., Grzelak P., Masajtis-Zagajewska A., Kaczmarska M., Stefanczyk L., Vermeer C., Maresz K., Nowicki M. (2015). Effect of vitamin K2 on progression of atherosclerosis and vascular calcification in nondialyzed patients with chronic kidney disease stages 3–5. Pol. Arch. Med. Wewn..

[85] Witham M.D., Lees J.S., White M., Band M., Bell S., Chantler D.J., Ford I., Fulton R.L., Kennedy G., Littleford R.C. (2020). Vitamin K supplementation to improve vascular stiffness in CKD: The K4Kidneys randomized controlled trial. J. Am. Soc. Nephrol..

[86] Oikonomaki T., Papasotiriou M., Ntrinias T., Kalogeropoulou C., Zabakis P., Kalavrizioti D., Papadakis I., Goumenos D.S., Papachristou E. (2019). The effect of vitamin K2 supplementation on vascular calcification in haemodialysis patients: A 1-year follow-up randomized trial. Int. Urol. Nephrol..

[87] Levy-Schousboe K., Frimodt-Møller M., Hansen D., Peters C.D., Kjærgaard K.D., Jensen J.D., Strandhave C., Elming H., Larsen C.T., Sandstrøm H. (2021). Vitamin K supplementation and arterial calcification in dialysis: Results of the double-blind, randomised, placebo-controlled RenaKvit trial. Clin. Kidney J..

[88] De Vriese A.S., Caluwé R., Pyfferoen L., De Bacquer D., De Boeck K., Delanote J., De Surgeloose D., Van Hoenacker P., Van Vlem B., Verbeke F. (2020). Multicenter randomized controlled trial of vitamin K antagonist replacement by rivaroxaban with or without vitamin K2 in hemodialysis patients with atrial fibrillation: The Valkyrie Study. J. Am. Soc. Nephrol..

[89] Holden R.M., Booth S.L., Day A.G., Clase C.M., Zimmerman D., Moist L., Shea M.K., McCabe K.M., Jamal S.A., Tobe S. (2015). Inhibiting the progression of arterial calcification with vitamin K in HemoDialysis patients (iPACK-HD) trial: Rationale and study design for a randomized trial of vitamin K in patients with end stage kidney disease. Can. J. Kidney Health Dis..

[90] Sabrina-Wong-Peixin Haroon B.-C., Tai L.-H.L., Lynette Teo A.D., Leon Schurgers B.-W.T., Priyanka Khatri C.-C.O., Sanmay Low X.-E.Y., Jia-Neng Tan S.S., Horng-Ruey Chua S.-Y.T., Weng-Kin Wong T.-W.-L. (2020). Treatment to reduce vascular calcification in hemodialysis patients using vitamin K (Trevasc-HDK): A study protocol for a randomized controlled trial. Medicine.

[91] Krueger T., Schlieper G., Schurgers L., Cornelis T., Cozzolino M., Jacobi J., Jadoul M., Ketteler M., Rump L.C., Stenvinkel P. (2014). Vitamin K1 to slow vascular calcification in haemodialysis patients (VitaVasK trial): A rationale and study protocol. Nephrol. Dial. Transpl..

[92] Roumeliotis S., Dounousi E., Salmas M., Eleftheriadis T., Liakopoulos V. (2020). Vascular Calcification in Chronic Kidney Disease: The Role of Vitamin K- Dependent Matrix Gla Protein. Front. Med..

[93] Van Ballegooijen A.J., Cepelis A., Visser M., Brouwer I.A., Van Schoor N.M., Beulens J.W. (2017). Joint association of low vitamin D and vitamin K status with blood pressure and hypertension. Hypertension.

[94] Dal Canto E., Beulens J.W., Elders P., Rutters F., Stehouwer C.D., van der Heijden A.A., van Ballegooijen A.J. (2020). The Association of Vitamin D and Vitamin K Status with Subclinical Measures of Cardiovascular Health and All-Cause Mortality in Older Adults: The Hoorn Study. J. Nutr..

[95] Van Ballegooijen A.J., Pilz S., Tomaschitz A., Grübler M.R., Verheyen N. (2017). The synergistic interplay between vitamins D and K for bone and cardiovascular health: A narrative review. Int. J. Endocrinol..

[96] El Borolossy R., El-Farsy M.S. (2021). The impact of vitamin K2 and native vitamin D supplementation on vascular calcification in pediatric patients on regular hemodialysis. A randomized controlled trial. Eur. J. Clin. Nutr..

[97] Roumeliotis S., Roumeliotis A., Eleftheriadis T., Liakopoulos V. (2021). Letter to the Editor regarding “Six months vitamin K treatment does not affect systemic arterial calcification or bone mineral density in diabetes mellitus 2”. Eur. J. Nutr..

[98] Roumeliotis S., Dounousi E., Eleftheriadis T., Liakopoulos V. (2019). Association of the Inactive Circulating Matrix Gla Protein with Vitamin K Intake, Calcification, Mortality, and Cardiovascular Disease: A Review. Int. J. Mol. Sci..

[99] Roumeliotis S., Roumeliotis A., Dounousi E., Eleftheriadis T., Liakopoulos V. (2021). Vitamin K for the treatment of cardiovascular disease in End-Stage Renal Disease patients: Is there hope?. Curr. Vasc. Pharm..

[100] Vlasschaert C., Goss C.J., Pilkey N.G., McKeown S., Holden R.M. (2020). Vitamin K supplementation for the prevention of cardiovascular disease: Where is the evidence? A systematic review of controlled trials. Nutrients.

[101] Kazzi N.J., Ilagan N.B., Liang K.-C., Kazzi G.M., Grietsell L.A., Brans Y.W. (1990). Placental transfer of vitamin K1 in preterm pregnancy. Obstet. Gynecol..

[102] Pucaj K., Rasmussen H., Møller M., Preston T. (2011). Safety and toxicological evaluation of a synthetic vitamin K2, menaquinone-7. Toxicol. Mech. Methods.

[103] Kaesler N., Schreibing F., Speer T., de la Puente-Secades S., Rapp N., Drechsler C., Kabgani N., Kuppe C., Boor P., Jankowski V. (2022). Altered vitamin K biodistribution and metabolism in experimental and human chronic kidney disease. Kidney Int..

